# Obesity is associated with shorter telomeres in 8 year-old children

**DOI:** 10.1038/s41598-019-55283-8

**Published:** 2019-12-10

**Authors:** Diana B. P. Clemente, Lea Maitre, Mariona Bustamante, Leda Chatzi, Theano Roumeliotaki, Serena Fossati, Regina Grazuleviciene, Kristine B. Gützkow, Johanna Lepeule, Dries S. Martens, Rosie R. C. McEachan, Helle M. Meltzer, Inga Petraviciene, Rémy Slama, Ibon Tamayo-Uria, Jose Urquiza, Marina Vafeiadi, John Wright, Tim S Nawrot, Martine Vrijheid

**Affiliations:** 1 ISGlobal, Institute for Global Health Barcelona, C/ Doctor Aiguader 88, 08003 Barcelona, Spain; 2 Center for Environmental Sciences, Hasselt University, Martelarenlaan 42, 3500 Hasselt, Belgium; 3 Universitat Pompeu Fabra, Plaça de la Mercè, 10, 08002 Barcelona, Spain; 4 CIBER Epidemiología y Salud Pública (CIBERESP), Av. Monforte de Lemos, 3-5, Madrid, Spain; 5 Bioinformatics and Genomics Program, Centre for Genomic Regulation (CRG), C/ Doctor Aiguader 88, 08003 Barcelona, Spain; 6 Department of Preventive Medicine, University of Southern California, University Park Campus, 90089-0911 Los Angeles, USA; 7 Department of Social Medicine, University of Crete, Andrea Kalokerinou 13, 715 00 Crete, Greece; 8 Department of Environmental Science, Vytautas Magnus University, Donelaičio 58, 44248 Kaunas, Lithuania; 9 Bradford Institute for Health Research, Bradford Royal Infirmary, Bradford, BD9 6RJ Bradford, UK; 10 Norwegian Institute of Public Health, P.O. Box 4404, Nydalen, NO-0403 Oslo Norway; 11 Inserm and University Grenoble-Alpes, U1209, IAB, Team of Environmental Epidemiology Applied to Reproduction and Respiratory Health, 110 Rue de la Chimie, 38400 Saint-Martin-d’Hères, France; 12 Department of Statistics, Faculty of Arts and Sciences, Harvard University, Cambridge, 02138 Massachusetts USA; 13 Department of Public Health & Primary Care, Unit Environment & Health, Leuven University, Oude Markt 13, 3000 Leuven, Belgium

**Keywords:** Predictive markers, Epidemiology, Risk factors

## Abstract

Telomere length is considered a biomarker of biological aging. Shorter telomeres and obesity have both been associated with age-related diseases. To evaluate the association between various indices of obesity with leukocyte telomere length (LTL) in childhood, data from 1,396 mother-child pairs of the multi-centre European birth cohort study HELIX were used. Maternal pre-pregnancy body mass index (BMI) and 4 adiposity markers in children at age 8 (6–11) years were assessed: BMI, fat mass, waist circumference, and skinfold thickness. Relative LTL was obtained. Associations of LTL with each adiposity marker were calculated using linear mixed models with a random cohort effect. For each 1 kg/m² increment in maternal pre-pregnancy BMI, the child’s LTL was 0.23% shorter (95%CI: 0.01,0.46%). Each unit increase in child BMI z-score was associated with 1.21% (95%CI: 0.30,2.11%) shorter LTL. Inverse associations were observed between waist circumference and LTL (−0.96% per z-score unit; 95%CI: −2.06,0.16%), and skinfold thickness and LTL (−0.10% per z-score unit; 95%CI: −0.23,0.02%). In conclusion, this large multicentric study suggests that higher child adiposity indicators are associated with short telomeres in children, and that associations are stronger for child BMI than for maternal pre-pregnancy BMI.

## Introduction

In adulthood, obesity has been associated with increased morbidity and mortality. Obesity can lead to oxidative stress and increased systemic inflammation^1,2^, both causes of telomere shortening in cells^3,4^. Telomeres are nucleoprotein structures containing tandem repeats of DNA (5′-TTAGGG-3′), situated at the end of the chromosomes^5^. Telomeres maintain the integrity of chromosomes, the stability of the genome, and prevent end-to-end chromosomal fusions^6^. When telomerase activity is lacking, DNA polymerase is unable to fully replicate the 3′ end of the DNA strand leading to telomere shortening in each cell division. Consequently, telomere length is known as a biomarker of biological aging. Individuals with shorter telomeres have with a higher risk for developing age-related diseases such as cardiovascular disease^7–9^, type 2 diabetes^10^ and increased mortality^11–13^. Both heritability and different environmental determinants have been linked with telomere length variability and attrition rate^14–18^. In studies with adult subjects, obesity and other pro-inflammatory risk factors have been associated with shorter leukocyte telomere length^17–21^. In children, however, case-control studies of telomere length and childhood obesity have brought discrepant results, with some showing that obesity is related to shorter telomeres^22,23^ but others finding no association^24^.

Recent findings have shown that telomere length at birth may be influenced by several intrauterine effects, such as air pollution exposure^25–30^. Additionally, Martens *et al*. (2016) showed that newborns with a mother with a higher pre-pregnancy BMI had shorter cord blood and placental telomeres^31^.

The rate of telomere attrition is greatest in young children^32^ followed by a slower attrition rate throughout adulthood^33^. Consequently, telomere loss in childhood is an important factor determining telomere length in adults, but little is known about the environmental exposures that impact on telomere attrition and molecular longevity during early life. The aim of the present study was to evaluate the effects of maternal pre-pregnancy BMI and child obesity parameters on telomere length measured in children approximately aged 8 years. These analyses were carried out in a multi-centre European birth cohort study in six different European countries.

## Results

### Characteristics of the study population

Table 1 describes the general characteristics of the study population (n = 1,396). The children had a mean (SD) age of 8 (1.5) years, 753 (53.9%) were boys, and they were mainly from white European origin (87.4%). The children had a mean (SD) BMI z-score of 0.48 (1.2) kg/m² and 15.4% and 6% were classified as overweight and obese, respectively. Mean (SD) maternal age at delivery was 30.5 (4.9) years and mean (SD) maternal pre-pregnancy BMI was 24.9 (5.1) kg/m² of which 853 (61.1%) had a normal weight. A total of 643 (46.1%) mothers were highly educated, 635 (45.5%) of the mothers were primiparous and 229 (13.4%) of the mothers actively smoked during pregnancy. The characteristics for the individual cohorts are presented in the Supplemental Materials (Table S1).

**Table 1:** General characteristics of the complete case study population.

	Mean ± SD or n (%)
**Children**	
Sex	
Girls	643 (46.1)
Boys	753 (53.9)
Ethnicity	
African	12 (0.9)
Asian	21 (1.5)
White European	1223 (87.5)
Mixed Native_American	13 (0.9)
Other	22 (1.6)
South-Asian	79 (5.7)
White_not European	26 (1.9)
Cohort	
INMA	428 (30.7)
MOBA	213 (15.2)
BIB	205 (14.7)
RHEA	202 (14.4)
KANC	199 (14.3)
EDEN	149 (10.7)
Age, years	8.0 ± 1.5
Relative telomere length^a^	1.0 (0.9–1.1)
BMI, z-score	0.5 ± 1.2
Normal	1097 (78.6)
Overweight	215 (15.4)
Obese	84 (6.0)
Fatmass^b^, z-score	7.1 ± 4.2
Waist circumference, z-score	0.025 ± 1.0
Skinfold thickness^c^, z-score	18.8 ± 9.2
**Mothers**	
Age at delivery	30.5 ± 4.9
Missing	15 (1.1)
Pre-pregnancy BMI, kg/m²	24.9 ± 5.1
Normal	853 (61.1)
Overweight	336 (24.1)
Obese	207 (14.8)
Education	
Low	219 (15.9)
Middle	480 (34.5)
High	643 (46.2)
Missing	54 (3.4)
Parity	
1	635 (45.5)
2	498 (35.7)
≥3	228 (16.3)
Missing	35 (2.5)

^a^Geometric mean and 25–75th percentile; ^b^measured from bioimpedence; ^c^sum subscapular and triceps thickness.

### Association between obesity parameters and telomere length

The association between maternal pre-pregnancy BMI and child BMI z-score, and child telomere length was linear (Supplemental Materials, Fig. S1). For each unit (1 kg/m²) increment in maternal pre-pregnancy BMI, child’s leukocyte telomere length was 0.26% shorter (95%CI: −0.48, −0.04%). Additionally, analyses using the maternal pre-pregnancy BMI as categorical variables showed that compared to children with mothers with a normal pre-pregnancy weight, telomere length was not shorter in children of mothers with pre-pregnancy overweight and obesity (Table 2).

**Table 2:** Obesity parameters and child’s leukocyte telomere length.

Mothers	n	% change	95% CI	P-value
Pre-pregnancy BMI	1396	−0.26	−0.48, −0.04	0.02
**Pre-pregnancy BMI categorical**				
Normal	853	Ref		
Overweight	336	−0.87	−3.56, 1.90	0.54
Obese	207	−2.20	−5.51, 1.23	0.21
**Children**	**n**	**% change**	**95% CI**	**P-value**
Fatmass z-score	1365	−1.03	−2.11,0.06	0.26
Waist circumference z-score	1373	−1.33	−2.38, −0.27	0.01
Skinfold z-score	1363	−0.17	−0.31, −0.05	0.005
BMI z-score	1396	−1.42	−2.28,−0.55	0.001
**BMI categorical**				
Normal	1097	Ref		
Overweight and obese	299	−3.14	−5.62, −0.60	0.02

Estimates are presented as a percentage change in average relative telomere length for each kg/m^2^ BMI increase in maternal pre-pregnancy BMI or for each z-score increase in the child’s anthropometric variable.

Models were adjusted for maternal education, maternal age at birth, child’s age, sex, qPCR batch, child’s ethnicity, maternal smoking during pregnancy and white blood cell type proportions.

Child’s telomere length was also inversely associated with obesity parameters measured in the children, including BMI, waist circumference, skinfold thickness, and fat mass (Table 2). Each unit increase in BMI z-score was associated with 1.42% (95%CI: −2.29, −0.55%) shorter telomere length. Inverse associations were observed between telomere length and waist circumference z-score (−1.33% per unit increase; 95%CI: −2.38, −0.27%), skinfold thickness z-score (−0.17% per unit increase; −0.31, −0.05%), and fat mass z-score (−1.03% per unit increase; 95%CI: −2.11, 0.06). Additionally, analyses using the obesity parameters as categorical variables showed that compared to children with a normal weight, telomere length was shorter in overweight and obese children (Table 2).

In a sensitivity analysis using the complete data we obtained exactly the same results as the analyses with the imputed data (data not shown). Additionally, we have included an analysis in which we stratified the associations for child BMI by maternal BMI group (Table 3). The estimates between child obesity indexes and telomere length at the age of 8 years was in the same order of magnitude in children of mothers with normal vs overweight and obesity. Maternal pre-pregnancy BMI and child BMI were weakly correlated with each other (r = 0.26, p < 0.001). Adding maternal pre-pregnancy BMI to the child BMI model and *vice versa* did not substantially change our reported associations for child BMI but did alter the associations for maternal BMI (Table 3). Stratification by sex in both models showed that the change in telomere length for each SD increase in BMI was in the same order of magnitude in boys and girls (Table 3). Finally, child weight was significantly associated with telomere length (−1.13% per z-score increment in child weight; 95% CI: −2.05, −0.21) (Table 3). No association was found between child height and telomere length (Table 3).

**Table 3:** Sensitivity analyses.

Child BMI	n	% change	95% CI	P-value
Overall	1396	−1.42	−2.28,−0.55	0.001
**By maternal pre-pregnancy BMI**				
Normal weight (BMI <25 kg/m²)	854	−1.25	−2.51, 0.02	0.05
Overweight and obese (BMI ≥25 kg/m²)	542	−1.58	−2.83, −0.31	0.02
**By sex**				
Male	753	−1.68	−2.80, −0.54	0.004
Female	643	−1.12	−2.47, 0.25	0.11
Adjusted for maternal pre-pregnancy BMI	1396	−1.24	−2.14,−0.32	0.008
**Child weight and height**				
Association between child weight and LTL^a^	1396	−1.13	−2.05, −0.21	0.02
Association between child height and LTL ^b^	1396	−0.14	−1.24, 0.97	0.80
**Maternal pre-pregnancy BMI**	**N**	**% change**	**95% CI**	**P-value**
Overall	1396	−0.26	−0.48, −0.04	0.02
**By sex**				
Male	753	−0.31	−0.60, −0.007	0.04
Female	643	−0.20	−0.52, 0.12	0.27
Adjusted for child BMI	1396	−0.15	−0.38,0.07	0.19

Estimates are presented as a percentage change in average relative telomere length for each kg/m^2^ BMI increase in maternal pre-pregnancy BMI or for each z-score increase in the child’s BMI.

^a^Estimates are presented as percentage change in average relative telomere length for each z-score increment in child weight.

^b^Estimates are presented as percentage change in average relative telomere length for each z-score increment in child height.

Models were adjusted for maternal education, maternal age at birth, child’s age, sex, qPCR batch, child’s ethnicity, maternal smoking during pregnancy and white blood cell type proportions.

## Discussion

The key finding of this study is that obesity parameters in children are associated with shorter leukocyte telomere length, independent of maternal education, maternal age, child’s age, sex, qPCR batch, child’s ethnicity, maternal smoking during pregnancy, and white blood cell type proportion.

Additionally, we showed that maternal pre-pregnancy BMI is associated with shorter telomeres in children. The findings of this study deserve attention because they indicate the possibility of premature aging due to exposures in early childhood. This study is, to the best of our knowledge, the largest by far in assessing associations between maternal and childhood obesity and childhood telomere length.

Previous case-control studies investigating telomere length and childhood obesity have found discrepant results. In a study of 148 Arab children, obese boys had a shorter mean telomere length compared with thin boys^23^, whereas, in a study of 53 Italian children telomere length of obese individuals did not differ from telomere length of non-obese individuals^24^. In another study conducted in 793 French children, mean telomere length was 24% shorter in obese children compared to that of non-obese children; however, when considering continuous BMI z-score, no association was observed^22^. All these studies were case-control design and so limited by selection bias.

In adults a meta-analysis suggests an inverse association between leukocyte telomere length and BMI^34^. In Chinese women, ages 40–70 years, BMI, waist circumference, and hip circumference were associated with shorter telomeres^35^. In a study containing 989 middle-aged individuals, telomere length and obesity parameters were negatively correlated^36^. In the Fels Longitudinal Study with 309 participants with ages from 8 to 80 years, BMI, hip circumference, waist circumference, visceral adipose tissue volume, and total body fat were all associated with shorter telomere length^37^. Furthermore, a study of Martens *et al*.^31^ showed that maternal pre-pregnancy BMI is associated with shorter placental and newborn cord blood telomeres. These findings help to explain the pre-pregnancy BMI effects on the new generation. In this paper we also looked at maternal pre-pregnancy BMI and child’s telomere length to see if the pre-pregnancy association observed by Martens *et al*. may persist into childhood. We found that leukocyte telomere length was consistently lower in children with mothers who had a higher pre-pregnancy BMI, although this association was attenuated when we added child BMI to the model.

The underlying mechanisms of the association between obesity and short telomeres still needs to be elucidated. Obesity is a condition that is defined as “abnormal or excessive fat accumulation that presents a risk to health”^38^. Obesity is a crucial factor in the management of the aging process in adipose tissue and then metabolic outcomes such as insulin resistance, cardiovascular disease, and diabetes^39–41^. A study of Minamino *et al*. found that in adipose tissue the p53 pathway, which is the fundament in adipose tissue aging and increased inflammation, can play an important role in the association between obesity and obesity-mediated aging^40^. This may be partially culpable for the observed inverse association with telomere length, as high levels of reactive oxygen species (ROS), produced by obesity, result in higher oxidative stress that is thought to quicken shortening of telomeres and cellular replication^4,42^. Oxidative stress causes single strand breaks in DNA in a direct and indirect way^4^. Compared to genomic DNA, telomeric DNA is relatively less capable of DNA repair. Furthermore, telomeres are an ideal target for oxidative damage because they contain G-rich fragments that are highly sensitive to ROS. Consequently, the higher level of oxidative stress induced by obesity leads to breakage of DNA and a faster decline in telomere length^43^.

The telomere loss in childhood may lead to increased risk for chronic diseases in adulthood. We were not able to estimate the effects of telomere loss based on absolute telomere length, since we used a qPCR method that cannot provide these absolute values. Nevertheless, an estimation based on available data from young adulthood telomere length would suggest an estimated loss of on average 8 kb^44^. This indicates that a decline of 1.21% leads to a loss of roughly 97 bp in leukocyte telomere length for each child BMI z-score unit increase. In adult leukocytes, the annual telomere loss was estimated between 32.2 and 45.5 bp^34^, indicating that each child BMI z-score unit increment is corresponding to a loss of 2.1 to 3.0 years (based on telomere attritions of 32.2–45.5 bp per year).

Our study has a number of potential limitations. Firstly, to determine telomere length we used a real-time PCR method, that has a higher assay variability in general in comparison with the traditionally used TRF method^45,46^. Nonetheless, an inter-laboratory comparison of our method showed that the coefficient of variation was less than 7%. Secondly, the assessment of telomere length at 8 year of age represents only a snapshot in childhood. We did not have data of telomere change throughout the entire pregnancy and the childhood period. Maternal telomere length might be a mediator of the association between pre-pregnancy BMI and child telomere length, as it is thought that overweight mothers can potentially have shorter telomeres. However, no data on maternal telomere length was available in our study to address this mediation. Thirdly, paternal age exerts a considerable effect on child telomere length^47^, however, this data was not available in our cohorts. Furthermore, Blood leukocyte is a mixed cell sample that would include granulocytes (neutrophils and eosinophils) as well as mononuclear cells. The effects of increased obesity parameters may be different in different cell types: average TL is shorter in neutrophils than lymphocytes and the halve life of neutrophils is much shorter than that of lymphocytes. However, in our analyses we adjusted for the proportion of the different white blood cell types: natural killer cells, B-cell, CD4T, CD8T, eosinophils, mononuclear cells, neutrophils. Finally, other factors that might influence child telomere length and that occur during pregnancy and childhood, like alterations of oxidative stress-related markers, were not measured.

In conclusion, this study suggests that higher BMI and higher adiposity indicators in children are associated with shorter telomeres in blood. Further, child BMI was more strongly associated with shorter telomere length than maternal pre-pregnancy BMI. This is the largest multicentric study to report associations between obesity parameters in mothers and children and telomere length in children. Telomere length in childhood predicts life expectancy; consequently, population-based cohort studies from birth onward are required to investigate if the difference in telomere length that we observe by maternal pre-pregnancy and childhood obesity status extends into adulthood. Furthermore, the directionality of the association between obesity and telomere length needs to be disentangled in future prospective studies.

## Materials and Methods

### Study population and data collection

The Human Early-Life Exposome (HELIX) study^48^ embodies a collective project across six established and continuing longitudinal population-based birth cohort studies in Europe: the Born in Bradford (BiB) study in the UK^49^, the Étude des Déterminants pré et postnatals du développement et de la santé de l’Enfant (EDEN) study in France^50^, the INfancia y Medio Ambiente (INMA) cohort in Spain^51^, the Kaunus cohort (KANC) in Lithuania^52^, the Norwegian Mother and Child Cohort Study (MoBa)^53^ and the RHEA Mother Child Cohort study in Crete, Greece^54^. The analysis of this paper made use of the HELIX subcohort. Inclusion criteria were: (a) children with age 6–11 years at the time of the visit, preferably ages 7–9 years if possible; (b) address history had to be available from birth to the last follow-up point. Finally we included mother-child pairs with complete clinical examination data and study-questionnaire, and available blood samples (n = 1396).

Local ethical committees approved the studies that were conducted according to the guidelines laid down in the Declaration of Helsinki. The ethical committees for each cohort were the following: BIB: Bradford Teaching Hospitals NHS Foundation Trust, EDEN: Agence nationale de sécurité du médicament et des produits de santé, INMA: Comité Ético de Inverticación Clínica Parc de Salut MAR, KANC: LIETUVOS BIOETIKOS KOMITETAS, MoBa: Regional komité for medisinsk og helsefaglig forskningsetikk, Rhea: Ethical committee of the general university hospital of Heraklion, Crete. Informed consent was obtained from a parent and/or legal guardian of all participants in the study.

Each cohort collected detailed information on maternal age at birth, maternal education, maternal marital status, smoking status during pregnancy, parity, and maternal ethnicity from each study participant during pregnancy or at birth by questionnaire or medical records^55^. The level of maternal education reported by the participant was used as the primary indicator of SES and categorized according to the International Standard Classification of Education (ISCED)^56^ as three levels: “low”, “middle” or “high”. Maternal smoking status was categorized as “no active smoking during pregnancy” and “active smoking during pregnancy”. Maternal ethnicity was defined for all cohorts and subdivided in 7 different groups (African, Asian, White European, mixed native-American, South-Asian, White- not European, or others). Perinatal parameters such as birth date, and newborn sex were obtained at birth.

### Blood collection, DNA extraction and white blood cells proportion

Buffy coat was collected in EDTA tubes. Leukocyte DNA was extracted using the Chemagen kit (Perkin Elmer) in batches of 12 samples. Samples were extracted by cohort and ultimately DNA concentration was determined in a NanoDrop 1000 UV-Vis Spectrophotometer (ThermoScientific) and with Quant-iT™ PicoGreen® dsDNA Assay Kit (Life Technologies).

Additionally, in samples of 1146 children white blood cell type proportions (CD4+ and CD8+ T-cells, natural killer (NK) cells, monocytes, eosinophils, neutrophils, and B-cells) were estimated from raw methylation data using Houseman algorithm^57^ implemented in the minfi R package^58^ and the Reinius reference panel^59^.

### Average relative telomere length measurement

Average relative telomere length was measured by a modified qPCR protocol as described previously^60^. Telomere and single copy-gene reaction mixture and PCR cycles used can be found in Martens *et al*.^31^. All measurements were performed in triplicate on a 7900HT Fast Real-Time PCR System (Applied Biosystems) in a 384-well format. On each run, a 6-point serial dilution of pooled DNA was run to assess PCR efficiency as well as eight inter-run calibrators to account for the inter-run variability. Relative telomere lengths were calculated using qBase software (Biogazelle, Zwijnaarde, Belgium) and were expressed as the ratio of telomere copy number to single-copy gene number (T/S) relative to the average T/S ratio of the entire sample set. We achieved CV’s within triplicates of the telomere runs, single-copy gene runs, and T/S ratios of 0.84%, 0.43%, and 6.4%, respectively.

#### Obesity parameters

Maternal anthropometrics included maternal pre-pregnancy BMI. Maternal height was measured and pre-pregnancy weight reported by the mother at the first trimester visit; these were used to calculate pre-pregnancy BMI (kg/m^2^). In adults, a BMI value within the range of 18.5–24.9 is categorized as normal, 25.0–29.9 as overweight and ≥30 as obese. In children from 5–19 years, a BMI-for-age value within the range of −2SD and +1 SD is categorized as normal, within +1 SD and +2 SD as overweight and ≥2 SD as obese^61^. Child’s anthropometrics included child’s BMI, waist circumference, skinfold thickness and fat mass^55^. Briefly, height (cm) and weight (kg) were measured using regularly calibrated instruments and converted to BMI age-and-sex standardized z-scores (zBMI) using the international World Health Organization (WHO) reference curves^61,62^. Waist circumference (cm) was measured as an indicator of visceral fat in duplicate to the nearest 0.1 cm with the child in a standing position, at the high point of the iliac crest at the end of a gentle expiration, with the use of a measuring tape (Seca 201; Seca Corporation). Skinfold thickness was measured at two anatomic sites (subscapular, and triceps) on the right side of the body in triplicate to the nearest 0.1 mm with a calibrated caliper following the protocols from the National Health and Nutrition Examination Survey III^63^. The sum of these two skinfolds was used in this study as an index of subcutaneous fatness. Measurements of bioimpedence were performed with the Bodystat 1500 (Bodystat Ltd, Douglas, Isle of Man) equipment after 5 minutes of lying down. The proportion of fat mass was calculated using published age- and race-specific equations validated for use in children^64^. Common standardized protocols and the same instruments were used for all measures across the cohorts. For the adiposity measures other than BMI we used the internal age and sex distribution of our entire study population to calculate age-sex-standardised z-scores.

### Statistical analysis

Continuous data were checked for normality. Average relative telomere lengths showed a skewed distribution and were log10 transformed to approximate a normal distribution. The obesity parameters were treated as both continuous (z-score unit) and categorical variables in models evaluating associations with telomere length. Generalized additive models (GAMs) were used to assess the linearity of the associations between the obesity parameters (maternal pre-pregnancy BMI, child’s BMI, waist circumference, skinfold thickness and fat mass) and telomere length. If the p-value for gain was higher than 0.05, the model was considered linear. Multivariable linear mixed models with a random cohort effect were used to address the different associations. All analyses were adjusted for *a priori* chosen covariates including maternal education, maternal age at birth, child’s age in days, sex, qPCR batch (2 categories), child’s ethnicity, maternal smoking during pregnancy, and white blood cell type proportions (% of natural killer cells, B-cell, CD4T, CD8T, eosinophils, mononuclear cells, neutrophils). We performed multiple imputation using chained equations to account for missing values of potential confounding variables, 20 datasets were generated and pooled for analyses (Supplemental Materials).

In a first sensitivity analysis we used the complete data instead of the imputed data for our initial analyses. In additional analyses we included in the child BMI model maternal pre-pregnancy BMI as a covariate. Similarly, we included child BMI as a covariate in the maternal pre-pregnancy BMI model. Further, we stratified our analysis of the associations between child BMI and leukocyte telomere length by maternal BMI group. We also stratified our BMI analyses by sex. Finally, the association between child weight and telomere length and the association between child height and telomere length were included.

All the mixed models were performed using the SAS 9.3 statistical software (SAS Institute Inc. Cary, NC, USA).

## Supplementary information

Supplemental materials

## Data Availability

The datasets used during the current study can be applied. The applications are received by the HELIX coordinator and are processed and approved by the HELIX project executive committee.
