# Supplementary material for: Obesity is associated with shorter telomeres in 8 year-old children
Source: Sci Rep. 2019 Dec 10;9:18739. doi: 10.1038/s41598-019-55283-8 (PMC6904465; doi:10.1038/s41598-019-55283-8)
Supplement: Supplementary file 1 — Supplemental materials [file 41598_2019_55283_MOESM1_ESM.docx]

**Supplemental Materials**

**Obesity is associated with shorter telomeres in 8 year-old children**

Diana BP Clemente, Lea Maitre, Mariona Bustamente, Leda Chatzi, Theano Roumeliotaki, Serena Fossati, Regina Grazuleviciene, Johanna Lepeule, Dries S Martens, Rosie R.C. McEachan, Helle Margrete Meltzer, Inga Petraviciene, Rémy Slama, Ibon Tamayo-Uria, Cathrine Thomsen, Jose Urquiza, Marina Vafeiadi, John Wright, Tim S Nawrot, Martine Vrijheid

**Table of contents:**

1. Supplemental Text S1. Additional information method: multiple imputation using chained equations…………………………………………………………………...pg.2
2. Supplemental Materials, Table S1………………………………………………….pg.3
3. Supplemental Materials, Figure S1…………………………………………………pg.5

**Supplemental Text S1. Additional information method: multiple imputation using chained equations**

We imputed exposures and confounders assuming missing at random (MAR) (Royston and White, 2011). The missing values for the potential confounders ranged from 0.2% (child´s ethnicity) to 15.6% (C-section). The missing values were imputed using the method of chained equations ^60^, using the mice package in R ^61^. We followed HELIX Statistical protocol for imputation process. The following points were taken into account 1) the imputation models included no more than 15 to 20 variables ^61^ and *quickpred* function was used to reduce the number of predictors; 2) predictive mean matching was used to imputed continuous covariates and logistic or multinomial regression was employed to impute binary or categorical exposures, respectively; 3) M=20 imputed datasets were created for each analyses ^60^. After imputation, we conducted diagnostics that included comparisons of the imputed and non-missing observations using density plots and strip-plots ^61^. If the variables included in the imputation process were not flagged and imputations seemed plausible, we included the predictors in the imputation model ^62^.

| **Supplemental Table S1.**  General characteristics of the complete case study population stratified by cohort | | | | | | |
| --- | --- | --- | --- | --- | --- | --- |
|  | **INMA (n = 428)** | **MOBA (n = 213)** | **BIB (n = 205)** | **RHEA (n = 199)** | **KANC ( n = 202)** | **EDEN (n = 149)** |
| **Children** |  |  |  |  |  |  |
| **Sex** |  |  |  |  |  |  |
| Girls | 206 (48.13) | 98 (46.0) | 93 (45.37) | 89 (44.72) | 92 (45.54) | 65 (43.6) |
| Boys | 222 (51.87) | 115 (54.0) | 112 (54.63) | 110 (55.28) | 110 (54.46) | 84 (56.4) |
| Missings | 0 (0.0) | 0 (0.0) | 0 (0.0) | 0 (0.0) | 0 (0.0) | 0 (0.0) |
| **Ethnicity** |  |  |  |  |  |  |
| African | 5 (1.17) | 0 (0.0) | 7 (3.41) | 0 (0.0) | 0 (0.0) | 0 (0.0) |
| Asian | 2 (0.47) | 6 (2.9) | 13 (6.34) | 0 (0.0) | 0 (0.0) | 0 (0.0) |
| White European | 380 (88.32) | 204 (95.7) | 89 (43.41) | 199 (100.0) | 202 (100.0) | 149 (0.0) |
| Mixed Native_American | 11 (2.57) | 2 (1.0) | 0 (0.0) | 0 (0.0) | 0 (0.0) | 0 (0.0) |
| Other | 4 (0.93) | 1 (0.4) | 17 (8.29) | 0 (0.0) | 0 (0.0) | 0 (0.0) |
| South-Asian | 0 (0.0) | 0 (0.0) | 79 (38.54) | 0 (0.0) | 0 (0.0) | 0 (0.0) |
| White_not European | 26 (6.07) | 0 (0.0) | 0 (0.0) | 0 (0.0) | 0 (0.0) | 0 (0.0) |
| Missings | 0 (0.0) | 0 (0.0) | 0 (0.0) | 0 (0.0) | 0 (0.0) | 0 (0.0) |
| **BMI, kg/m²** | 18.1 (3.0) | 16.4 (1.9) | 16.0 (2.0) | 16.8 (2.6) | 16.4 (2.3) | 17.9 (2.9) |
| Missings | 0 (0.0) | 0 (0.0) | 0 (0.0) | 0 (0.0) | 0 (0.0) | 0 (0.0) |
| **Gestational age** | 39.9 ± 1.4 | 40.1 ± 1.7 | 39.7 ± 1.8 | 38.4 ± 1.4 | 39.4 ± 1.3 | 39.8 ± 1.7 |
| Missings | 0 (0.0) | 6 (2.8) | 0 (0.0) | 0 (0.0) | 5 (2.5) | 0 (0.0) |
| **Age at mtDNA content and telomere lenght assessment, years** | 9.02 ± 0.65 | 8.5 ± 0.5 | 6.6 ± 0.2 | 6.5 ± 0.3 | 6.5 ± 0.5 | 10.8 ± 0.6 |
| Missings | 0 (0.0) | 0 (0.0) | 0 (0.0) | 0 (0.0) | 0 (0.0) | 0 (0.0) |
| **Mothers** |  |  |  |  |  |  |
| **Age at delivery** | 31.5 ± 4.2 | 32.8 ± 3.7 | 28.6 ± 5.8 | 30.9 ± 4.8 | 28.57 ± 5.0 | 30.7 ± 5.0 |
| Missings | 1 (0.2) | 6 (2.8) | 1 (0.5) | 2 (1.0) | 2 (1.0) | 0 (0.0) |
| **Pre-pregnancy BMI** | 23.9 (4.7) | 22.6 (3.1) | 28.3 (5.3) | 24.2 (4.5) | 27.8 (5.3) | 23.4 (4.3) |
| Missings | 0 (0.0) | 0 (0.0) | 0 (0.0) | 0 (0.0) | 0 (0.0) | 0 (0.0) |
| **Education** |  |  |  |  |  |  |
| Low | 99 (23.1) | 0 (0.0) | 88 (42.9) | 9 (4.5) | 12 (5.9) | 11 (7.4) |
| Middle | 174 (40.7) | 41 (19.2) | 31 (15.1) | 111 (55.8) | 69 (34.2) | 55 (36.9) |
| High | 141 (32.9) | 164 (77.0) | 64 (31.2) | 79 (39.7) | 116 (57.4) | 83 (55.7) |
| Missings | 14 (3.3) | 8 (3.8) | 22 (10.7) | 2 (1.0) | 5 (2.5) | 3 (2.0) |
| **Active smoking during prenancy** |  |  |  |  |  |  |
| Yes | 109 (225.46) | 9 (4.4) | 25 (12.2) | 43 (21.6) | 13 (6.44) | 31 (20.8) |
| No | 311 (72.66) | 198 (91.6) | 157 (76.6) | 156 (78.4) | 184 (91.09) | 118 (79.2) |
| Missings | 8 (1.87) | 9 (4.4) | 23 (11.2) | 1 (0.5) | 5 (2.5) | 0 (0.0) |
| **Parity** |  |  |  |  |  |  |
| 1 | 230 (53.7) | 93 (43.7)) | 83 (40.5) | 74 (37.2) | 84 (41.6) | 71 (47.7) |
| 2 | 165 (38.6) | 86 (40.4) | 52 (25.4) | 85 (42.7) | 59 (29.2) | 51 (34.2) |
| ≥3 | 28 (6.5) | 28 (13.1) | 56 (27.3) | 35 (17.6) | 54 (26.7) | 27 (18.1) |
| Missings | 5 (1.2) | 6 (2.8) | 14 (6.8) | 5 (2.5) | 5 (2.5) | 0 (0.0) |
| Continuous covariates expressed by mean and standard deviation (SD); categorical covariates described by number and frequencies (%). | | | | | | |

**
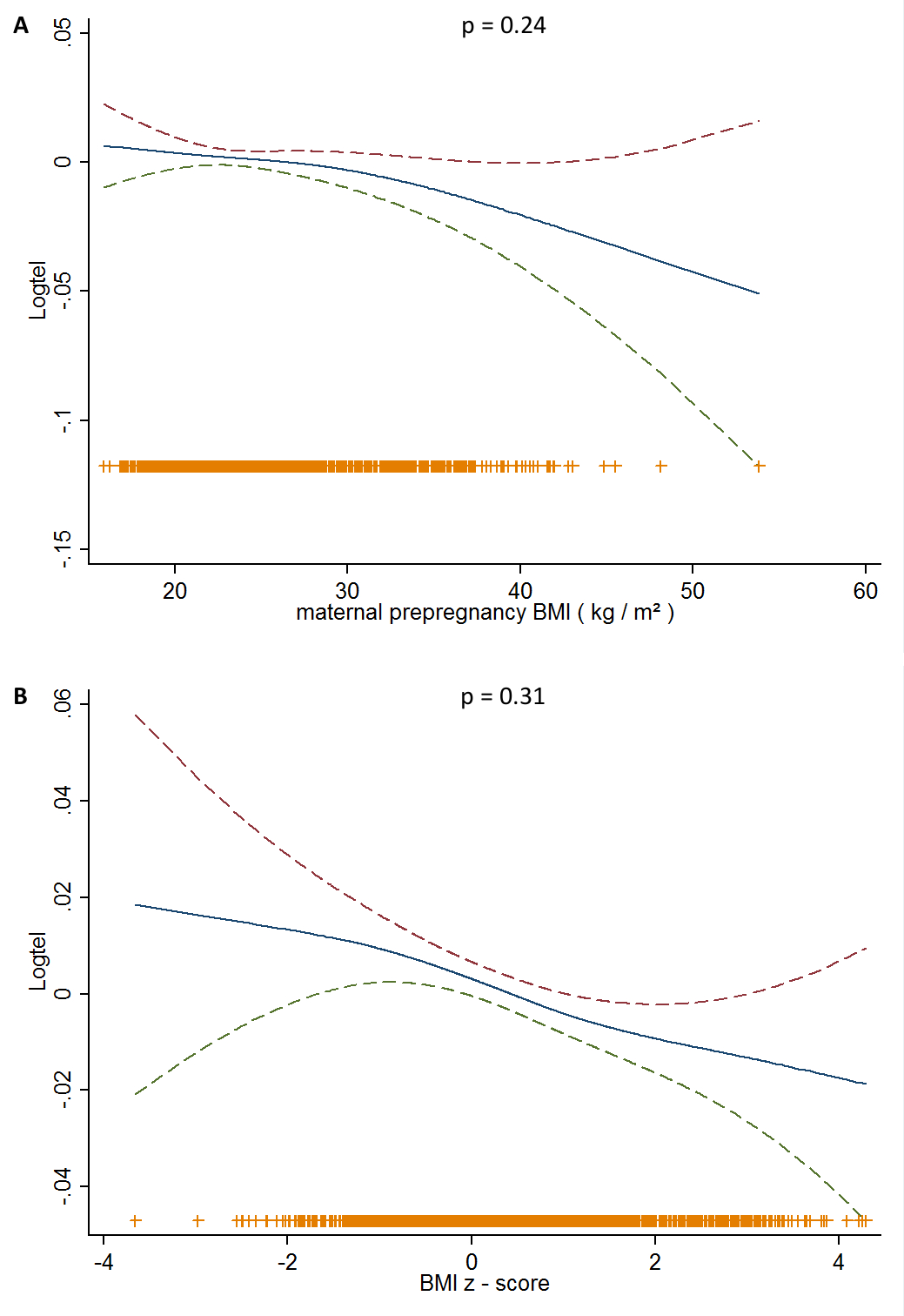
**

**Supplemental Figure S1.** GAM models show the linear relation between (A) maternal pre-pregnancy BMI and child leukocyte telomere length and (B) child BMI z-score and child leukocyte telomere length. P-value = p-value for gain. Models were adjusted for maternal education, maternal age at birth, child’s age, sex, qPCR batch, child’s ethnicity, maternal smoking during pregnancy and blood cell type proportions.
